# Barriers and facilitators of male engagement in Community Client-Led Antiretroviral therapy Delivery groups (CCLADS) for HIV care and treatment in Southwestern Uganda: a qualitative study

**DOI:** 10.1186/s12913-022-07544-y

**Published:** 2022-01-29

**Authors:** Jovaile Kushemererwa, Moses Muwanguzi, Esther C. Atukunda, Halimah Nantambi Kikomeko, Odwee Ambrose, Denis Androdri, Brillian Kembabazi, Josephine Nambi Najjuma

**Affiliations:** 1 Faculty of Medicine, Mbarara University of Science and Technology, Box, 1410 Mbarara, Uganda; 2 Department of Public Health, School of Allied Health and Sciences, Kampala International University Teaching Hospital, Box, 71 Bushenyi, Uganda

**Keywords:** CCLADs, Male engagement, ART access, Uganda

## Abstract

**Background:**

Male engagement in HIV programs is a persistent challenge that results in poor utilization of HIV care services. Differentiated service delivery models, such as Community Client-Led Antiretroviral Delivery Groups (CCLADs), provide an opportunity for male engagement in HIV care. In southwestern Uganda. In southwestern Uganda few men living with HIV (MLWHIV) are involved in CCLADS. We aimed to identify facilitators, barriers and perceptions to CCLADs enrollment by MLWHIV at ART Clinics in southwestern Uganda.

**Methods:**

A qualitative study was conducted among MLWHIV who were registered and receiving ART at two ART Clinics/health facilities in southwestern Uganda, irrespective of their enrollment status into CCLADs. In-depth interviews (IDI) were conducted among recruited HIV positive men, and Key informant interviews (KIIs) among clinic in-charges and counselors, women enrolled in CCLADS using a semi-structured interview guide. We used thematic analysis to analyze the data from the interviews.

**Results:**

We conducted 16 interviews, 7 KII and 8 IDI were conducted. MLWHIV and key informants shared the facilitators and barriers. Men who were not involved in CCLADs shared the barriers to joining the CCLADs. The themes identified included 1. Motivations to join CCLADS 2. Challenges related to CCLADS initiation 3. Perceived facilitators for male participation in CCLADS, 4. Perceived barriers for male participation in CCLADS and 5. Proposed strategies for best implementation of CCLADs for better male engagement. Overall men liked the idea of CCLADs but they had preferences on how they should be implemented.

**Conclusion:**

Men’s enrollment into CCLADs is still low despite the benefits. Addressing the barriers to men’s engagement and adopting proposed strategies may improve men’s enrollment in CCLADS and thus improve their access to ART, Adherence and quality of life.

## Introduction

Antiretroviral therapy (ART) improves the quality of life of Persons Living with HIV (PLWHIV) [1] Through lowering the viral load, improving the individual’s immunity and reducing the chances of getting opportunistic infections. This is achieved through better ART access and adherence.

In 2016, WHO endorsed the Differentiated Service Delivery Models (DSDM) program which was adapted in Uganda as a means to improve the health seeking behavior of PLWHIV in terms of access to treatment [2]. These groups are formed voluntarily by the people in the same community with the same interests and goals [3]. They consist of 4 to 6 people and must meet a certain criterion. The criteria include; been on ART for at least 4 years, viral suppression (less or equal to 1000 copies/ml of blood), no substance abuse and not on TB medication. The CCLADs are aimed at giving adherence support, psychological support, solidarity and medication refills [4].

DSDM for receiving ART have been proposed and implemented in Uganda. These include facility-based delivery and community-based delivery models. Among the community delivery models, Community Client ART Delivery Model (CCLADs), or known as Community ART Groups (CAGs) or Community ART Refill groups (CARGS) have been reported to improve male engagement and access to ART [3]. Of these, Community Client-Led ART Delivery (CCLAD) model is said to be more effective in improving adherence and retention of men in care and enhance patient’s self-management [3]. In sub-Saharan Africa (SSA), suboptimal male engagement in HIV programs is still a persistent challenge, for example, in 2013, 88% of Kenyan pregnant women tested for HIV compared to 4.5% of their male partners [5]. This has led to lower utilization of HIV care services, such as HIV testing, prevention and treatment services, which worsens treatment outcomes for men [3]. The Population-based HIV surveys show that men consistently lag behind women in awareness of their HIV status, adherence to ART and are less likely to link to HIV care to maintain viral load suppression [6, 7]. Men also interact less with the health system than women resulting in poor clinical outcomes; addressing the gender gap in HIV testing and treatment access is required for successful ART scale up in SSA [5, 8, 9].

CCLADS model is comprised of 3 to a maximum of 12 stable ART members living in the same community. Pregnant women, breastfeeding women, infants, children and adolescents are not eligible to join these groups. They are encouraged to use the facility-based model since they need continuous monitoring. Members pick up ARVs at the health facility in turns and distribute them amongst themselves. Each member visits the facility for at least twice in a year for clinical assessment. They manage their own health and share experiences about living positive with HIV. Information regarding their health is recorded on the CCLAD monitoring form and taken to a health facility during the next refill, to update their facility records. Each member of the group must visit a health facility twice a year for a comprehensive clinical assessment [10].

CCLAD groups is one of the DSD subgroups are said to improve adherence & retention of PLWH in HIV care, resulting in good treatment outcomes [11]. The Commissioning for Quality and Innovation (CQUIN) showed that only 6% of HIV patients nationally are actively participating in CCLAD groups of which majorities are women [12]. This has been attributed to, lack of awareness, misunderstanding of how these groups operate, stigma, confidentiality concerns, and few perceived benefits of participation [5, 13]. However, with the low percentage of the general population enrolling into the CCLADS, the proportion of men enrolled in these groups compared to the women is still very low [12]. Stigma, poverty, judgmental health care services, the perception that clinics are places for women are some of the reasons why men are reportedly staying away from health facilities [5, 14].

Health facilities in Sheema district, southwestern Uganda are some of the facilities with a high-volume HIV patient that adapted CCLADS model 1 year ago. However, these facilities still don’t know why few men are enrolling into these groups in Sheema district southwestern Uganda. Men play a very crucial role in the transmission of HIV, for successful implementation and scale-up of ART delivery in SSA strategies must address the gender gap in treatment access [5]. This study, therefore sought to describe perceptions, facilitators, and barriers of men living with HIV towards enrolling in CCLADS.

## Methods

### Study design

This qualitative study used an exploratory descriptive approach to understand the perceptions of men towards CCLADS, barriers and facilitators to MLWHIV enrollment into CCLADS. This was carried out from April to May 2021.

### Study setting

This study was carried out in Sheema District southwestern Uganda at two purposively selected health facilities; Kabwohe Health Center IV and Shuuku Health Center IV. Whereas Sheema district has three high volume HIV government funded clinics, all received training on how to implement CCLADS but only Kabwohe and Shuuku Helath Centr IV were implementing CCLADS at the time of the study. A health center IV is a mini hospital that is headed by a medical officer. Both health facilities are government-funded. At these health facilities, participants choose the model of ART delivery they prefer, either facility-based or community-based, after CCLADS had been instituted for 4 months. Following this introduction, clients who have been receiving medication in Community refill groups are being transformed to CCLADS. The health facility staff advise individuals from the same area who meet the criteria for joining CCLADS, to form a group where one individual comes to the clinic to collect the ART for the rest of the group members. Both health facilities provide general health care, including inpatient and outpatient. The Clinics serve clients who come from all the districts in southwestern Uganda. Most of the individuals at these clinics serve are Banyankole and the most common language is Runyankole (Local language).

Kabwohe Health center IV has a total of 2305 clients with 151(6.5%) pediatric clients, and 34.6% of the adult clients are Males. Six hundred twenty-nine of these men have been on ART for more than two years, only 7 of these men were on TB treatment and 10 men had CD4 less than 350mls. The ART clinic takes place on Monday, Wednesday and Thursday, the Monday clinic is dedicated to pregnant mothers and the others are general clinics. The second health facility (Shuuku Health center IV), ART clinic takes place on Tuesday and Thursday. The ART clients choose to either be part of those that receive their ART either at the facility or in the community. At the clinic, they can still choose whether to receive the ART as a group or as an individual.

### Study participants

We collected data among the MLWHIV receiving ART care from both health facilities who were 18 years and above since infants, children and adolescents are not eligible to join CCLADS (Ministry of Health implementation Guide for differentiated service delivery models of HIV). We included adults who did not have a known mental health diagnosis, had no speech problems and were stong enough to participate in the interview. These men were either participating (for facilitators) or not participating (for barriers) in the CCLADS. These were selected by help of the counselors at the Clinic. The other participants were key informants that included women who were in CCLADS (because they have better access to care than men), the facility incharges, peer educators and community health workers. We only interviewed respondents who were stable enough to respond to the interview questions.

### Data collection procedures

In-depth Interview (IDI) guides were developed by the research team basing on the previous literature in English and translated to Runyankole before the interviews. Study participants were purposively selected, we included only those that fit the inclusion criteria. These interviews took place in person (Face-to-face) at the clinic by male research assistants. We choose to work with male research assistants so that the MLWHIV who were the participants in IDI could be more comfortable to share. The same research assistants were trained to interview the females study participants. The research assistants were experienced qualitative researchers and were trained on the research protocol. Appointments for the interview were made via phone call by the research assistants.

Interviews were audio-recorded using digital voice recorders, which were stored on a password protected laptop, and backed up on an external flash disc. Audio files were transcribed verbatim in the language they were conducted in Runyankole (Local Language) or English by the interviewer. Runyankole transcripts were later translated to English by another research assistant and checked by the interviewer for clarity and content translation.

We carried out 16 interviews. The topics discussed in the IDIs were aimed to understand the barriers and facilitators of men joining CCLADs, reasons for joining or not, experiences in joining CCLADS, advantages of joining CCLADS, strategies for male participation in CCLADS. Data were collected by three male middle-aged research trained research assistants. We considered the age and gender of the research assistants to make sure the participants are comfortable to share. Data was collected until when the saturation point was reached [15].

### Ethical consideration

Ethical approval was obtained from Mbarara University the Research and Ethics committee MUREC 28/11–20. Administrative permission to carry out the study at the facility was sought from from Sheema District Health Officer (DHO), and health facility Medical in-charges. Before data collection was started the purpose of the research was explained to the participants by the research assistants. The informed consent included explanations about the purpose and objectives of the study, the benefits, and the risks that could accrue from the study. Unique identification numbers were indicated on the data collection tools for privacy and anonymity. All recordings were stored on password-protected computers only accessed by the research team members. All participants were given a chance to ask questions and/or seek further clarification about the study. After understanding the informed consent, the participants signed or put a thumbprint the informed consent form.

### Data analysis

Data analysis was done in an iterative process starting from the first interview. The transcripts were read and re-read immediately after transcription and the process continued throughout the study. English transcripts were read by three research team members (JN, MM, JNN) to get familiar with the data, and later codes were developed; similar responses were clustered to form sub themes which later informed the main themes of the study. Data were analyzed manually using thematic analysis. We present the findings in the result section by participants number with participant number, form of interview and date.

## Results

We conducted 16 interviews, 7 In-depth interviews and 9 Key informant interviews. Participants of IDI were all MLWHIV receiving ART services *at Kabwohe and Sheema HC IVs. Their characteristics are summarized in* Table 1*.* Key informants included those that had a better understanding of the functioning of the health facilities and ART delivery systems, the summary of their characteristics are summarized in Table 2. These included women who were receiving ART through CCLADS, Counselors, ART clinic nurses and the facility in charges.

**Table 1:** Characteristics of IDI participants

Participant	Age in years	Mode of ART delivery
1	45	CCLADs
2	51	CCLADS
3	35	CLLADs
4	40	Individual facility based
5	50	CCLADS
6	41	Individual Facility based
7	39	CCLADS

**Table 2:** Characteristics of KI participants

Participant	Gender	Age	Role
1	Male	35	Peer leader
2	Female	37	Nurse
3	Female	60	CCLAD Member
4	Female	33	CCLAD Member
5	Female	23	CCLAD Member
6	Male	30	Counselor
7	Female	32	CCLAD member
8	Female	30	Clinic in-charge
9	Male	33	Peer leader

### Emerging themes

Findings from the thematic analysis yielded five broad themes. 1. Motivations to join CCLADS 2. Challenges related to CCLADS initiation 3. Perceived facilitators for male participation in CCLADS, 4. Perceived barriers for male participation in CCLADS and 5. Proposed strategies for best implementation of CCLADs for better male engagement. These are summarized in Table 3;

**Table 3:** Summary of results

Theme	Components
Motivations to join CCLADS	– Reduced transport costs – Reduced waiting hours at the hospital – Reduced stigma – Encouragement by other men – Poor attitude by health workers
Challenges related to CCLADS initiation	– Number of people in the same area – Lack of Trustworthiness – Drug abuse – Fear for stigma – Funds to facilitate the CCLADs
Perceived facilitators for male participation in CCLADS	– Incentives – Income-generating activities – Other reasons to make a group rather than ART delivery – Voluntary formation
Perceived barriers for male participation in CCLADS	– Information gap – Fear of HIV disclosure – Secrecy and community perceptions
Proposed strategies for best implementation of CCLADs for better male engagement	– Closing the information gap – Snowballing to find eligible members

#### Motivations to join CCLADS

The study participants shared current challenges with the facility-based ART delivery and Individual community-based ART delivery systems as these are some of the drivers to join the CCLADs. These challenges ranged from the long waiting hour in the above modes to the transport costs, and stigma that occurs when individuals find you at the clinic in the clinic waiting area. Participants mentioned that CCLADS may solve these. Participants mentioned that CCLADs reduce the frequency of going to the facility whereby one of the group members is the one who goes to the facility and the rest just contribute a certain amount of money which he uses as transport to and from the hospital hence saving money as one of our participants told us that,

“*They have helped us most of the time before we used to go and receive medicine every month, so where do you get such transport, but after introducing these groups, you go there once in 6 months, and you still keep receiving your medicine….” .P8-IDI-04/2021*

Another member of CCLADS mentioned that;

“*We were coming from a village far away and everyone was using like 15000shs for transport to come to the facility, so we discovered that if we are 6 people, you find that we have spent about 65000shs, so we decided to give that money to one person, 15000shs, so that he can come to the facility and receive for us our medicine” P1-IDI-04/2021*

The money saved from the transport expenditure is then used to give help with other basic needs. Also, men articulated that instead of all of them waiting for the ART at the health facility only one of them does. This saves them time to do other income generating activities thus encouraging them to use CCLADs as a mode of ART delivery. Since only one of the group members is responsible for picking the medicine for the rest of the other members. The rest of the members when it is not their turn get enough time to do the other jobs.

“*Some people in this village have their gardens that they cultivate, so you might find that on the day when he is supposed to go to the clinic, he can’t leave his garden… so one person goes to the hospital and the rest stay in their gardens” P11-IDI-2021*

Male peers encourage each other in HIV care and management. They have to call each other to remind themselves to take their medicines and also organize meetings where they come together and which makes them feel that they are not the only ones taking ART medicine

*“…so it helps you in always remembering to take your medicine, your fellow members will always remind you to swallow your medicine” P3-IDI-04/2021*

Also, the unfriendly approach to the HIV positive men while receiving ART. Female Nurses were reported to have some bad attitudes towards the HIV-positive men accessing the HIV care services especially when receiving the ART medications and this is attributed to too much workload arising from high clinic turn ups of the patients at the facility. This makes men to join these groups to reduce on the frequency they interact with the nurse. One of the participants reported that,

*“… sometimes when you go to pick your medicine from the nurse, she talks to you badly( rudely) and you feel so small(lowers my self-esteem)…” P5-IDI-04/2021*

#### Challenges related to CCLADS initiation

Health care providers at both clinics emphasized that they have some clients who are not permanent residents of these areas. Also, they mentioned that some clients come from further districts/distances to get ART since they don’t want to disclose their HIV status in their home area. Both clinics had clients who travel for approximately 32 km or more to the clinic. In such cases these individuals could not form a group since it was only one person from a given community. These individuals leave HIV clinics in their home area/ districts or pass by other HIV clinic and access care at these clinics. This is because of the perceived stigma from the people in their home areas.

Thus, the fear of the stigma that might arise from the community members was reported as a challenge to CCLADs initiation. The majority of the participants reported that once they join these groups, these groups will be used as identifiers for HIV positive people and yet the community has a perception that everyone who is HIV positive is a promiscuous. This was strongly emphasized by one of the study participants by;

*“There are some who decide to hide so that no one knows that they are HIV positive so when you tell them to join these associations then if people know that one of them is HIV positive, then they will know that it is an association of HIV patients and take him to be a prostitute” p5- IDI-4/2021*

Also, participants who used Alcohol reported that they were not allowed to join the CCLADs. Because of alcoholism these individuals were identified as not eligible to join the CCLADS even though their other parameters/requirements were meeting the inclusion criteria.

This is because men in the community are looked at as heavy drinkers, and in the process, they end up forgetting to take their medication or even take the ART medications together with the alcohol resulting in drug interactions hence increasing the viral load as one of the participants said that;

“*Those who take alcohol are not allowed to join these groups because if you take alcohol your viral load can be high or you can easily forget to take your ART medication, so nurses cannot allow you to join.” P3-IDI-04/2021*

#### Perceived facilitators for male participation in CCLADS

Participants also identified items or strategies that would facilitate men to join CCLADS. These included giving them incentives in terms of non-monetary terms after joining CCLADS. Other participants identified having other activities linked to groups on top of the ART delivery as a facilitator for male participation. For example, having an economic activity that they are all interested in, would help them meet and work together to feel a sense of belonging to the team. These economic activities would not only give them a sense of belonging but also would work as a cover to those they don’t wish to share their HIV status with.

On the other hand, some of the key informants stated that they had facilitated the formation of CCLADs but there were not operating as expected. This was because they were just trained on how these groups operate and not given the materials to use such as the registers and also money for facilitation while mobilizing the HIV positive men to form the groups, as the counselors from one of the facilities uttered out that;

“We *were supposed to form those clubs in 2020, but we got some challenges with the people that were supporting us in calling people for sensitizations and to join those clubs. I think their facilitation was lacking, so we put everything to a hold” KI-IDI-4/2021*

Voluntary formation of the groups by the men; These groups are formed by 4 to 6 people in the community staying near to each other and of the same interest in the community. These interests include being involved in the same economic activities, or meeting to watch or participate in games. And always have meetings where they sit to discuss the issues arising and also encourage each other to perform their daily activities. This was confirmed by one of the participants who said,

“*We formed the groups ourselves, eh… we were given knowledge then we formed the groups ourselves, we knew that so and so will be able to stay in our group. The doctor did not form these groups for us, he just gave us knowledge on how to form them” P5-IDI-04/2021*

But this was faced with the challenge of not know who is eligible to join. Non-disclosure of the HIV statuses among the men in the village was identified as a challenge to forming CCLADS. Participants mentioned that they have challenges in identifying fellow men who are HIV positive except those that they meet at the health facility where they receive their ART. Since they get ART on different days and some of them get their ART medication from far facilities which might make them think that they are the only infected men in their communities

*“We don’t know ourselves in the village so you find that there are some people who are infected with HIV in this village but hide themselves, some of them instead go to Kitagata (Further health facility) so such people don’t join these associations” P7-IDI-14/2021*

#### Perceived barriers for male participation in CCLADS

HIV positive men’s awareness of the CCLADs was mentioned as a barrier to their participation. Most of the study participants were aware of the existence of these groups, but not satisfied with the operation of these groups, whereby only the group member is supposed to go and pick the medication on their behalf hence limiting them a chance to interact with the health facility as more frequently as they wish. One of the participants cited that;

*“HIV infected people that are in the same village gather and form an association and you chose a leader who will keep going to the hospital to pick HIV medicine on your behalf and yet someone would need counselling that is at the clinic” P5-IDI-04/2021*

On the other hand, other participants who were not involved in CCLADS said that they had never heard about the existence of these groups and that’s why they are not into these groups. One of the participants pointed out that,

*“……hmmm as for me, I have never heard about these groups…..actually they will help us a lot!“P4-IDI-04/2021*

This was supplemented to by the confidentiality concerns whereby misunderstandings may arise within the group members that may result in one of them disclosing a member’s personal health identifying information to the public and it gets to know the person’s status.

On the other hand, CCLADS are being seen as a threat to marriage and sexual relationships. It was mentioned that once a man tests positive, he does not wish his wife and children to know as this may result in segregation, fear and depression among the family members especially if he finds out that he is the only HIV positive person. This was strongly supported by one of our participants who said,

“*There is when you find that you are taking HIV medication and you don’t want your wife to know about it because if she learns of it she will quarrel” P5-IDI-04/2021*

### Masculinity amongst the men

It was noted that most men whether part of the group or not would wish to keep their HIV status confidential. Sometimes they do this to avoid the misappropriation of blame between husband and wife. It was reported that most men never want to be looked at as the cause of any problem in the family and thus they did not want to access HIV care or join CCLADS for the fear to their HIV status to be known to others. To emphasize the point about men’s fear to HIV testing, one of the participants said;

“…. *First of all, men are the ones that usually spread HIV, whenever his wife tells him to go for an HIV test, the man tells her that, “go for it first” P8-IDI-04/2021*

#### Proposed strategies for best implementation of CCLADs for better male engagement

However, participants gave in their views on how best these groups can be implemented to enhance male enrollment into ART delivery groups and these were;

Providing information to men so that they are aware of all the available options and their advantages. Participants proposed that this information should be made at the lowest level of administration where groups are expected to be formed. They proposed that this could be done in village meetings or at the village health center, as one of the participants emphasized that,

*“When they bring seminars, they should also conduct them on the health center one so that even men who have not yet come here at this health center IV can also go for checkups and also receive their HIV medicine which will make them join these groups…” P6-IDI-04/2021”*

Other participants proposed snowballing among the HIV positive men to get participants in the CCLADS, whereby the individual in the group passes on the information to the fellow HIV man who interns informs the other HIV positive man to inform the other about the benefits of these groups and encourage them to join the groups

*“There are some men that I know that used to get medicine from here and those ones I am capable of talking to them and explain to them the benefits which will encourage them to join these associations or we can even add them to ours and also tell them to bring their friends to join us” P2-IDI-04/2021*

The provision of incentives in form of monetary terms, was also mentioned as a way of improving men’s involvement in CCLADS. This would be in form of transport refund. Some of the key informants reported that the provision of monetary support would make the CCLADs appealing to men. This money would be used to start income-generating projects such as poultry and also provide transport to group members who come to collect the ART medication as this could change the community perceptions toward these groups. One of the clinics in-charges echoed that,

*“The biggest thing would be that, if you can help us with funds so that, even if we don’t give the person receiving medicine money, your funds are available for transport for him to use, that would be very helpful” P3-IDI-04/2021”*

## Discussion

The study determined the barriers, facilitators and perceptions of MLWHIV towards joining CCLADS in Sheema district southwestern, Uganda. To our knowledge, this study is among the first studies carried out evaluating male enrollment into CCLADS in Uganda since most of the studies only focus on retention of HIV positive men in care, [16] and access to the effectiveness of the CAD groups [11].

Although information about CCLADs is available at the health facilities and frequently shared at the clinics, knowledge of how CCLADS operate was the major barrier to male enrollment. This finding confirms that men are poor health seekers and also interact with the health facility less frequently which is a persistent challenge [8]. Lack of information about health services has long been cited as the common barrier to men’s healthcare utilization behavior worldwide [11] and also contribute to the low intake of these new models [17, 18]. In the study carried out in South Africa, the participants were still confused about the criteria and procedure to join these groups [18]. Therefore, better marketing of these groups needs to be done not only at the HIV clinics but also at the community level.

A major key motivator for MLWHIV enrollment was voluntary formation of these groups. Whereby MLWHIV choose people of the same interest and form a group. This is in agreement with the study carried out in Zimbabwe where most of the participants suggested flexibility in the formation of these groups such as men only groups motivated them to join the groups. This approach would build participant ownership and partial reassurance that co-members would guard confidentiality [3]. Additionally, the provision of funds for income generating projects and some materials to the ART department may increase the number of MLWHIV that enroll in these programs. Studies about male engagement in HIV peer support groups have found out that income-generating activities may motivate men to be involved in the HIV programs [19]. This would help men have some funds for other use and hospital access. It has been noted that poverty directly affects male engagement due to lack of resources like transport [5]. A study carried out in Malawi found out that income generating activities were initiated and highly valued yet they were not part of the formal structure of CCLADS [20]. Therefore, helping men start an income generation activity as a CCLAD group might improve their access to ART.

This study adds to the growing literature on male engagement in HIV care, by providing and in-depth explanation of the impact of the rapidly expanding CCLAD groups, hence provides information to the policymakers on how best these groups can be modified to have a large number of men enrolling into them.

Since this program is not yet integrated into all ART clinics in Uganda, we could only assess the two health facilities in the district where it was implemented. This qualitative study describes the only perceptions, barriers and facilitators but not the extent/Impact of CCLADS. We recommend a multi-site quantitative study to assess the impact of CCLADS on male engagement, and adherence.

## Conclusion

Active CCLADs may solve the gender disparities in ART access. Addressing the barriers to male enrolment in CCLADS may encourage enrolment and engagement leading to improved satisfaction and enhancing treatment outcomes for men. To achieve this, targeted seminars that clearly explain what the CCLADs are, how they function and how to join should be extended to all men in their workplaces and communities. Easy to read materials like charts and financial literacy support to create money generating activities for men may also increase enrollment and retention in care. Certain programs such as CCLADs that aim at community-based stigma reduction should be emphasized however there are some men prefer facility based and provide a more confidential environment than the CCLADs. No one is likely to meet the preferences of all the recipients of this program and countries must think wide to decide how best they can implement these programs to satisfy the needs of their citizens.

### Study limitations

One of the limitations was done in one district in southwestern Uganda, the results may not reflect the experience of men’s engagement in CCLADS in other settings. Whereas we collected data on the participants’ roles in the groups, we did not assess the perceptions of the participants towards these roles in the groups. Also data on how the COVID-19 pandemic affected the CCLADs was not collected.

## Data Availability

Data and research materials used in the current study are available from the corresponding author on reasonable request.

## Abbreviations

ART: Antiretroviral Therapy
CAG: Community ART groups
CCLADS: Community Client Lead ART Delivery Groups
DSDM: Differentiated service delivery modalities
WHO: World Health Organization
HC IV: Health Center Four
HIV: Human Immunodeficiency Virus
SSA: Sub-Saharan Africa
UNCST: Uganda National Council of Science and Technology
MUST: Mbarara University of Science and Technology
MLWHIV: Men Living with HIV
IDI: In-Depth Interviews
KII: Key Informant Interviews
